# Epidemiologic and clinical investigations during a chikungunya outbreak in Rio Grande do Norte State, Brazil

**DOI:** 10.1371/journal.pone.0241799

**Published:** 2020-11-20

**Authors:** Joelma D. Monteiro, Joanna Gardel Valverde, Ingryd Camara Morais, Cassio Ricardo de Medeiros Souza, João Ciro Fagundes Neto, Marília Farias de Melo, Yasmin Mesquita Nascimento, Brenda Elen Bizerra Alves, Leandro Gurgel de Medeiros, Hannaly W. Bezerra Pereira, Anne Aline Pereira de Paiva, Diego G. Teixeira, Márcia Cristina Bernardo de Melo Moura, Alessandre de Medeiros Tavares, José Veríssimo Fernandes, Selma M. B. Jeronimo, Josélio M. G. Araújo

**Affiliations:** 1 Immunogenetics Laboratory, Institute of Tropical Medicine, Federal University of Rio Grande do Norte, Natal, Brazil; 2 Post-Graduate Program in Parasite Biology, Biosciences Center, Federal University of Rio Grande do Norte, Natal, RN, Brazil; 3 Laboratory of Molecular Biology for Infectious Diseases and Cancer, Department of Microbiology and Parasitology, Biosciences Center, Federal University of Rio Grande do Norte, Natal, RN, Brazil; 4 Laboratory of Virology, Institute of Tropical Medicine, Federal University of Rio Grande do Norte, Natal, Brazil; 5 Post-Graduate Program in Health Sciences, Health Sciences Center, Federal University of Rio Grande do Norte, Natal, RN, Brazil; 6 Laboratory of Complex Diseases, Institute of Tropical Medicine, Federal University of Rio Grande do Norte, Natal, Brazil; 7 Institute of Science and Technology of Tropical Diseases, Brazil; 8 Secretaria Municipal de Saúde, Centro de Controle de Zoonoses, Natal, Brazil; Beni Suef University, Faculty of Veterinary Medicine, EGYPT

## Abstract

The first autochthonous case of chikungunya virus (CHIKV) infection in Brazil was in September 2014 in the State of Amapá, and from there it rapidly spread across the country. The present study was conducted in 2016 in the state of Rio Grande do Norte, and the aims were to describe the epidemiological and the clinical aspects of the CHIKV outbreak. Biological samples from 284 chikungunya suspected cases were screened for CHIKV and Flavivirus (FV) RNA using qRT-PCR. Negative PCR samples were also screened for anti-CHIKV and anti-FVIgM by ELISA. CHIKV RNA were detected in 125 samples mostly occurring from January through March (46%), mainly affecting adults and older adults. We found a gradual decrease in viral RNA over the disease time. Anti-CHIKV IgM was found in 47.5% after negative CHIKV qRT-PCR. Interestingly, 45.0% simultaneously had positive results for CHIKV and FV IgM, suggesting the occurrence of virus co-circulation. The most frequent symptom was fever (91%). Women presented more chance to develop nausea and abdominal pain compared to men. Our data described and allows us to better understand the clinical and epidemiological aspects of the 2016 chikungunya outbreak in Rio Grande do Norte and can help in the early clinical diagnosis of the virus.

## Introduction

Chikungunya virus (CHIKV) was first isolated in 1952 in Tanzania, Africa [1, 2]. CHIKV is a member of the *Togaviridae* family, *Alphavirus* genus, and has a positive-sense, single-stranded RNA genome of approximately 11,8kb [3].

The first autochthonous cases of CHIKV in Brazil were confirmed in Oiapoque, Amapá State, in September 2014. A week later, autochthonous cases were also confirmed in Feira de Santana, in Bahia state [4]. The Oiapoque cases were caused by the Caribbean genotype of the virus, while the Feira de Santana cases corresponded to the East-Central/South African Genotype (ECSA) [5]. The ECSA genotype was frequently found in CHIKV autochthonous cases diagnosed in the northeast and west-central regions of Brazil [6]. Brazil reported 263,598 cases of chikungunya fever (CF), 145,059 (55.0%) of which were confirmed cases diagnosed between January 3^rd^ to December 10^th^ of 2016, with an incidence rate of 128.9 cases/100,000 inhabitants. The outbreak was widespread, as cases were reported in 2,752 out of the 5,570 (49.4%) municipalities of Brazil. The northeast region of the country reported the highest incidence rate of 405.2 cases/100,000 inhabitants, and the state of Rio Grande do Norte reported an incidence of 718.5 cases/100,000 inhabitants [7].

Chikungunya fever is an acute and usually self-limiting febrile illness which can affect anyone at any age. CHIKV infection confers long term immunity, thus the epidemic peaks tend to decrease as the population’s immunity improves [8]. Signs and symptoms last longer than 2 to 4 days. Not all infected individuals develop symptomatic disease, but symptomatic cases usually start with an abrupt onset of high fever (> 38.9°C), followed by myalgia, arthritis, and generalized arthralgia which is often disabling for patients [9]. Polyarthralgia and polyarthritis are usually bilateral, symmetrical, and more frequently occur in the hands, wrists, interphalangeal joints, feet and ankles, but can also affect shoulder and knee joints [9]. Periarticular swelling can also be observed [9, 10]. A maculopapular rash and facial edema are described in 40 to 50% of CHIKV patients [11]. Children frequently present abullous rash with pronounced sloughing as signs and symptoms, in addition to petechiae and gingivororrhage. Ocular involvement may also occur as an atypical manifestation, with recovery in about six to eight weeks [12, 13]. Most of the clinical symptoms may last a few weeks; however, polyarthralgia can persist months or even years in about 30 to 40% of infected individuals [14, 15], with neurological outcomes accounting for up to 25% of atypical cases, and up to 60% of severe atypical cases [16].

Herein, we describe the epidemiological, immunological and clinical aspects of the CHIKV infection during the 2016 epidemic in the state of Rio Grande do Norte, Brazil, considering clinical and laboratory data of 284 suspected cases of CHIKV infection.

## Methods

### Clinical samples

Whole blood, serum, plasma, cerebrospinal fluid, urine and/or blister fluid were collected from suspected cases in Rio Grande do Norte State, Brazil. Samples were tested in the Laboratory of Infectious Diseases and Cancer at the Federal University of Rio Grande do Norte. One sample was collected from each patient. Patient information, name, gender, age, address, sample collection date, onset of symptoms, as well as description of signs and symptoms were recorded on data sheets which accompanied the samples. The samples were stored at -70°C until use.

### Ethics statement

Ethical clearance was obtained with the approval resolution number CAAE 51057015.5.0000.5537 from the Ethics Committee in Research of the Federal University of Rio Grande do Norte. Participant consent was waived by the ethics committee. All samples were anonymized.

### Climate data

Rainfall data from January to December 2016 were obtained from the weather stations operated at the Federal University of Rio Grande do Norte, Natal.

### Viral RNA extraction and reverse transcription followed by quantitative real-time polymerase chain reaction (TaqMan^®^ system)

Viral RNA was extracted using a QIAmp Viral Mini Kit (Qiagen, Inc., Valencia, USA), following the manufacturer’s instructions. RNA were amplified by one-step quantitative real-time PCR (qRT-PCR). All samples were first screened for CHIK using CHIKV 6856F (500 nM) and CHIKV 6981R (500 nM) primers and CHIKV 6919P (100 nM) probe, as previously described [17]. The samples that were qPCR negative for CHIKV were screened for FV (Dengue virus serotypes 1–4 (DENV)) and zika virus (ZIKV). Next, zika_qRT_F (200nM), zika_qRT_R (200 nM) primers and zika_qRT_P (125nM) probe were used for ZIKV screening, as previously described [18]. Amplification was conducted using ABI Prism 7500 Fast. The cycle threshold (CT) values of qRT-PCR was used to estimate viral load. A lower CT value means higher estimated viral load. The Ct value is the cycle number at which the fluorescence generated within a reaction crosses the fluorescence threshold, a fluorescent signal significantly above the background fluorescence. The Ct is inversely proportional to the original relative expression level of the gene of interest. The nested RT-PCR protocol for DENV detection and typing was performed as previously described [19]. Positive cases were considered as those which presented a positive qRT-PCR.

### Anti-CHIKV and anti-FV IgM ELISA

High affinity Costar plates 3590 (Corning Inc., New York, USA) were sensitized with 100ng of CHIKV E2, 20ng of FV (ZIKV, DENV1-4) NS1 antigens (Meridian Life Science, Memphis, USA) overnight at 4°C. The plates were blocked with 1% PBS-Tween^®^20 buffer. The serum samples of suspected cases were diluted in 0.1% PBS-Tween^®^ buffer to order of 1:400 and added to the plate. Horseradish peroxidase (HRP)—conjugated anti-human IgM (Rockland, Limerick, USA) was added to order of 1:10000. The reaction was revealed using 3,3,5,5–tetramethylbenzidine (TMB) as substrate (SeraCare, Milford, USA) and read at 450nm wavelength.

The cut-off was calculated as the mean of 3 negative control samples plus three times the standard deviation with 95% of confidence interval (CI), as recommended [20]. Results were expressed as relative optical density (rOD), which is the ratio between the optical density (OD) sample and the plate cut-off. The results were considered positive when rOD ≥ 1.1; negative when rOD < 0.9; and undetermined when 1.1 > rOD ≥ 0.9 (undetermined zone).

Validation of anti-CHIKV and anti-FV IgM ELISA protocols was performed by Receiver Operating Characteristic (ROC) analysis. ROC statistics, with sensitivity and specificity values, are available in Supporting Information (S1 Fig and S1 Table).

### Statistical analysis

Cycle Threshold (CT) values detected by qRT-PCR and relative optical density (rOD) values determined by ELISA are presented as medians. All graphs and statistical tests were performed using Graph Pad 6.0 Prism Software (La Jolla, California). The distribution normality of samples was determined by the D’Agostino-Pearson Amnibus test. Statistical significance was determined using the Two-tailed Student’s *t-*test. The Chi-squared/Fisher’s exact tests were used to evaluate the association between gender and chikungunya fever symptoms. P values < 0.05 were considered statistically significant.

## Results

### Epidemiologic characteristics of confirmed CHIKV cases

**Fig 1:**
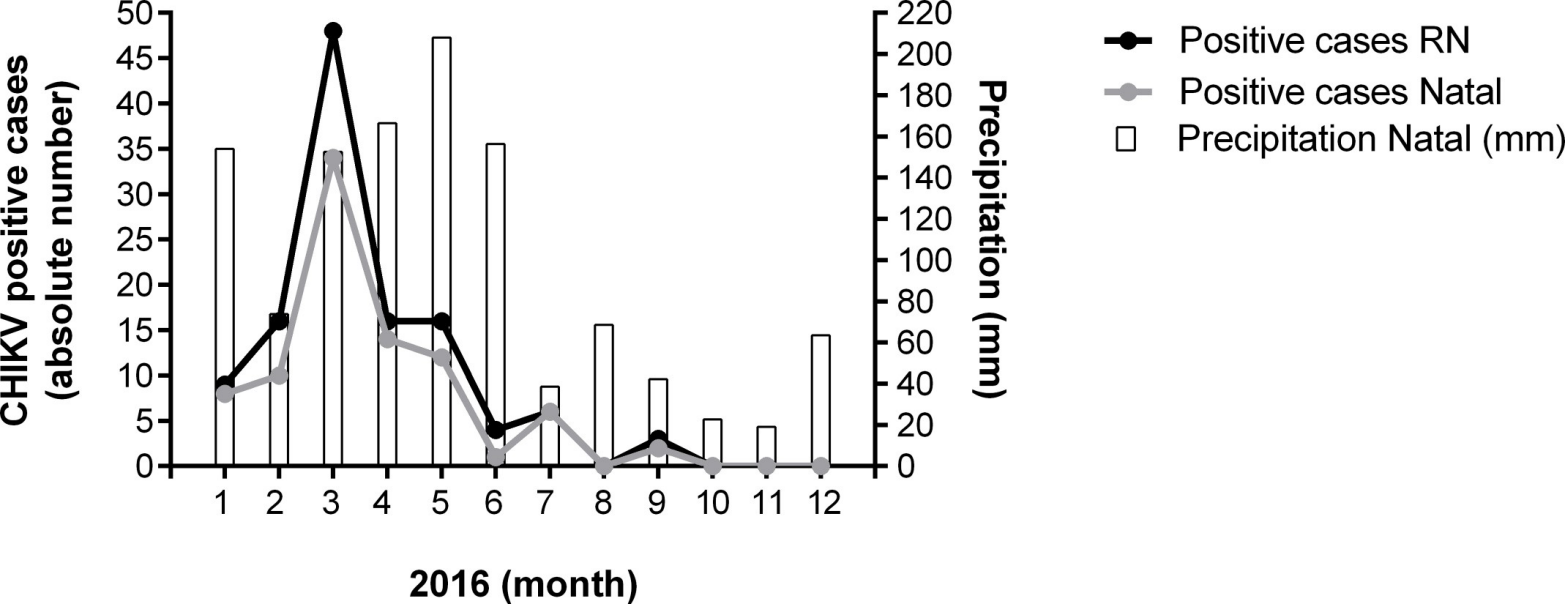
Positivity for chikungunya virus and precipitation. Absolute number of positive chikungunya cases in Natal and Rio Grande do Norte and precipitation in Natal per week from January 2016 to December 2016.

A total of 284 suspected CHIKV cases from Rio Grande do Norte state were tested by qRT-PCR from January to December 2016. One hundred twenty-five (44.4%) were confirmed for CHIKV. Negative samples for CHIKV qRT-PCR were also negative for FV qRT-PCR. The largest number of positive cases occurred in March (48 cases), decreasing thereafter with case detection until September (Fig 1). Among 125 CHIKV positive cases, 87 occurred in Natal city (70%). The rainfall index gradually increased after February and remained high until June, then decreased thereafter. The peak of chikungunya cases was observed in March, followed by a decline during the rainy season (Fig 1).

### Frequency of CHIKV infection by gender and age

Among 125 positive qRT-PCR chikungunya cases, the frequency was higher in females (52%), followed by males (42.4%) and neonates (5.6%) (Table 1). Among females, 9.2% were pregnant (n = 6). In addition, although more positive female Chikungunya cases were found, the male gender showed 2 times more chance to present a positive PCR compared to female: odds ratio (OR): 2.01; 95% confidence interval (CI): 1.16–3.47, p = 0.0121.

**Table 1:** Demographic characteristics of chikungunya cases, Rio Grande do Norte, Brazil, 2016.

	Suspected CHIKV cases	Positive CHIKV cases
**Gender**	**N (%)**	**N (%)**
Male	93 (32.7)	53 (42.4)
Female	158 (55.6)	65 (52.0)
*Pregnant females	15 (9.5)	6 (9.2)
Neonates	33 (11.6)	7 (5.6)
Age (years)**		**N (%)**
Years (mean±SD)		35 ± 24
Neonates	33 (11.6)	7 (5.6)
Children (30 days <1 year)	17 (6.0)	10(8.0)
1–10	15 (5.3)	7 (5.6)
11–20	32 (11.3)	14 (11.2)
21–30	32 (11.3)	11 (8.8)
31–40	35 (12.3)	17 (13.6)
41–50	30 (10.6)	20 (16.0)
51–60	16 (5.6)	8 (6.4)
>61	31 (10.9)	20 (16.0)
Not informed	43 (15.1)	11 (8.8)

Positive cases stratified by gender, neonates and age group.

*Percentage among female gender.

Positive CHIKV cases aged from neonates to 88 years old and the mean age was 34 years old. The absolute number of CHIKV positive cases in adults aged 41 to 50 years, and adults older than 61 years old was higher compared to the other age groups (20) (Table 1). Both age groups showed a higher positivity among the suspected cases (S2 Fig).

### Frequency of CHIKV RNA detection among biological samples

**Fig 2:**
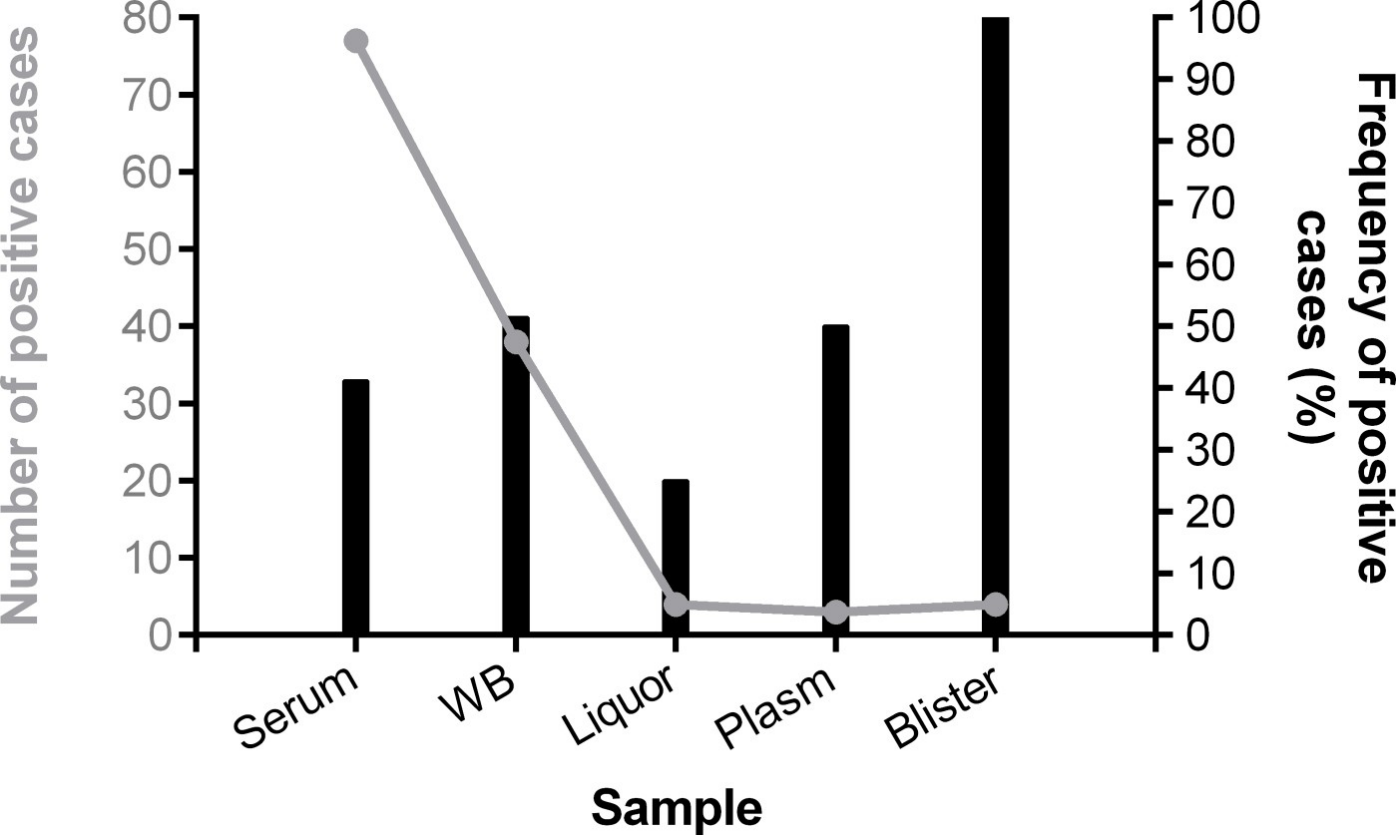
Chikungunya virus detection in different sample types. Absolute number and frequency of chikungunya virus per sample type, from Rio Grande do Norte, Brazil, 2016.

Different types of samples (serum, plasma, cerebrospinal fluid, blister and whole blood [WB]) were tested. However, only serum or WB were available in most cases. The fluid obtained from blisters presented the highest chance to show positive qRT-PCR compared to serum, plasma, WB or cerebrospinal fluid (odds ratio (OR): 11.96; 95% confidence interval (CI): 0.637–224.4, p = 0.0355). However, the positivity was higher in the serum samples (*n* = 77; 41.2%), and the frequency was higher in the blister samples (*n* = 4; 100%) (Fig 2).

### Association of time after symptom onset and CHIKV viremia

**Fig 3:**
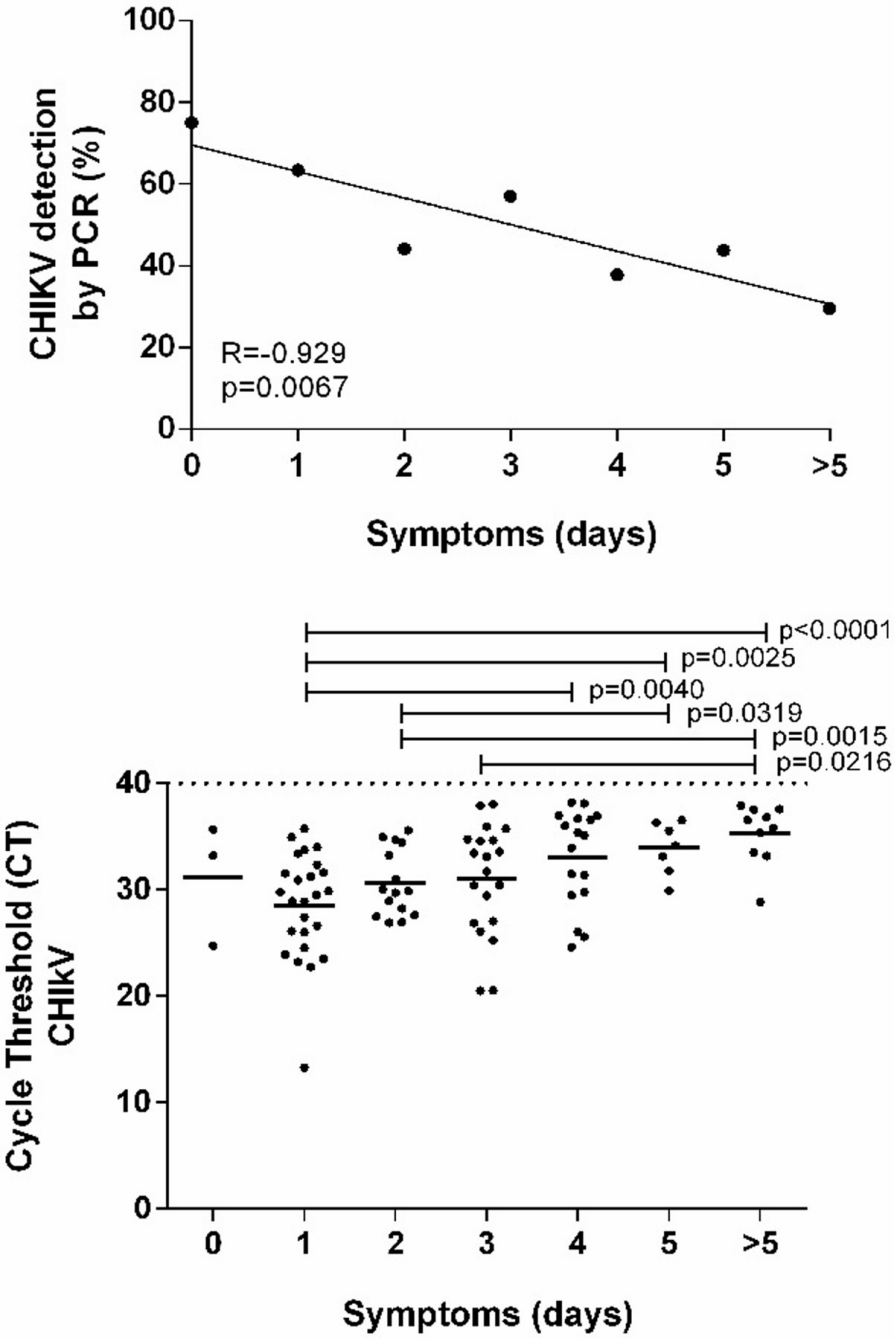
Chikungunya virus detection during a chikungunya outbreak, Rio Grande do Norte, Brazil, 2016. Percentage of chikungunya virus detection correlated to day of symptom onset (A) and estimated blood viral loads of chikungunya on different days after the onset of symptoms (B).

We found an inverse correlation of days of symptom onset and percentage of positive molecular diagnosis for CHIKV (R = -0.929; p = 0.006) (Fig 3A). Among positive samples, the Cycle Threshold (CT) in each day of symptoms is shown in Fig 3B. The CT was higher closer to the onset of clinical symptoms (median: 28.56) and decreased thereafter (median: 35.31; p<0.0001) (Fig 3B).

### Frequency of anti-CHIKV and anti-FV IgM detection among negative CHIKV RNA samples

**Fig 4:**
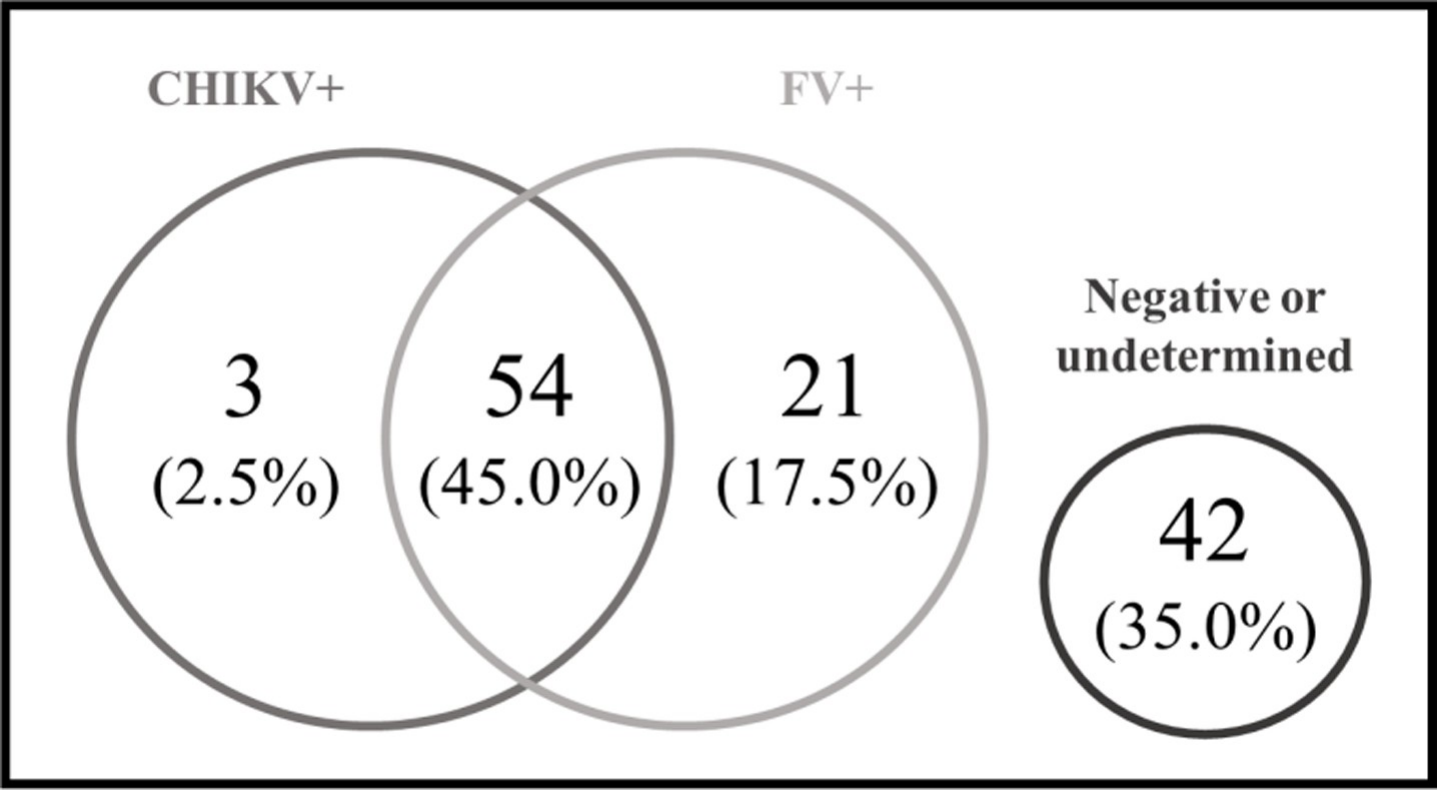
Venn diagram of IgM positivity. Venn diagram for 120 negative arbovirus quantitative polymerase chain reaction (qPCR) showing chikungunya virus and flavivirus IgM positivity during chikungunya outbreak, Rio Grande do Norte, Brazil, 2016.CHIKV+: anti-CHIKV IgM positive samples; FV+: anti-ZIKV and/or anti-DENV1-4 IgM positive samples.

A total of 120 negative CHIKV qRT-PCR samples were tested to identify anti-FV IgM and/or anti-CHIKV. CHIKV IgM was detected in 57 serum samples (47.5%), while 63 (52.5%) were negative or undetermined. Among the CHIKV IgM negative or undetermined samples, 21 (21.7% of total samples) had positive results for FV (Fig 4). Interestingly, 54 samples (45.0% of total samples) presented CHIKV and FV IgM. Moreover, 42 samples (35.0%) presented negative or undetermined results for all tested antigens.

### Signs and symptoms of CHIKV suspected cases

Among suspected cases, the qRT-PCRN negative CHIKV samples showed a higher chance to not present signs and symptoms typical of CHIKV infection compared to those which were qPCR positive (Odds ratio: 3.3, p<0.0261). The clinical characteristics of the suspected cases were similar except for fever, arthralgia and conjunctivitis which were more common among the positive CHIKV compared to negative CHIKV (Odds ratio respectively: 3.8, p<0.0001; 2.7, p = 0.0003; 3.6, p = 0.0027).

### Signs and symptoms of positive CHIKV RNA cases

The symptoms among positive CHIKV cases were ranked from the most to the less frequent (Table 2), however neonates were excluded from this analysis. The most frequent symptom was fever (91%). The symptoms which presented less than 5% of notification were grouped in “other signs and symptoms” (blister, neurological manifestations, anorexia, dysgeusia, mucosal bleeding, dry cough, leukopenia, plaquetopenia and lymphadenopathy). Females presented a 2.6-fold more chance to develop nausea and a 3.7-fold more chance to develop abdominal pain compared to males (Table 2). Although skin blisters are a common sign found in neonates infected with CHIKV (S3 Fig), four adults presented skin blistering (3.2%). None of the positive CHIKV presented anemia, hypotension, seizures, hepatomegaly, splenomegaly, ascites, cyanosis, stroke, jaundice, dyspnea, pleural effusion, respiratory insufficiency or brain death.

**Table 2:** Chikungunya symptoms frequency and gender in Rio Grande do Norte, Brazil, 2016.

Signs and symptoms	Symptom frequency (%)	Male symptom frequency (%)	Female symptom frequency (%)	Odds ratio	Confidence interval	p-value
Fever	91.0	90.9	91.1	1.020	0.257–4.049	1.000
Arthralgia	86.0	81.8	89.3	1.852	0.591–5.803	0.386
Myalgia	61.0	63.6	58.9	0.820	0.364–1.849	0.683
Arthritis	56.0	47.7	62.5	1.825	0.819–4.070	0.160
Headache	56.0	54.5	57.1	1.111	0.502–2.461	0.841
Back pain	55.0	47.7	60.7	1.693	0.762–3.762	0.228
Exanthema	48.0	43.2	51.8	1.413	0.639–3.127	0.426
Nausea	47.0	34.1	57.1	2.578	1.138–5.841	0.027*
Edema	45.0	45.4	44.6	0.968	0.438–2.140	1.000
Asthenia	24.0	27.3	21.4	0.727	0.290–1.827	0.638
Vomiting	21.0	18.2	23.2	1.360	0.508–3.647	0.625
Conjunctivitis	21.0	25.0	17.9	0.652	0.248–1.714	0.461
Abdominal pain	19.0	9.1	26.8	3.659	1.117–11.980	0.038*
Retro orbital pain	18.0	9.1	25.0	3.333	1.011–10.990	0.065
Vertigo	18.0	11.4	23.2	2.358	0.770–7.220	0.190
Diarrhea	12.0	9.1	14.3	1.667	0.467–5.945	0.542
Itching	12.0	4.5	17.9	4.565	0.945–22.060	0.062
Photophobia	9.0	4.5	12.5	3.000	0.591–15.240	0.292
Othersignsandsymptoms	18	2.02	2.33	0.870	0.340–2.228	0.812

*Increased chance for females to develop chikungunya symptoms compared to males (p<0.05).

**Fig 5:**
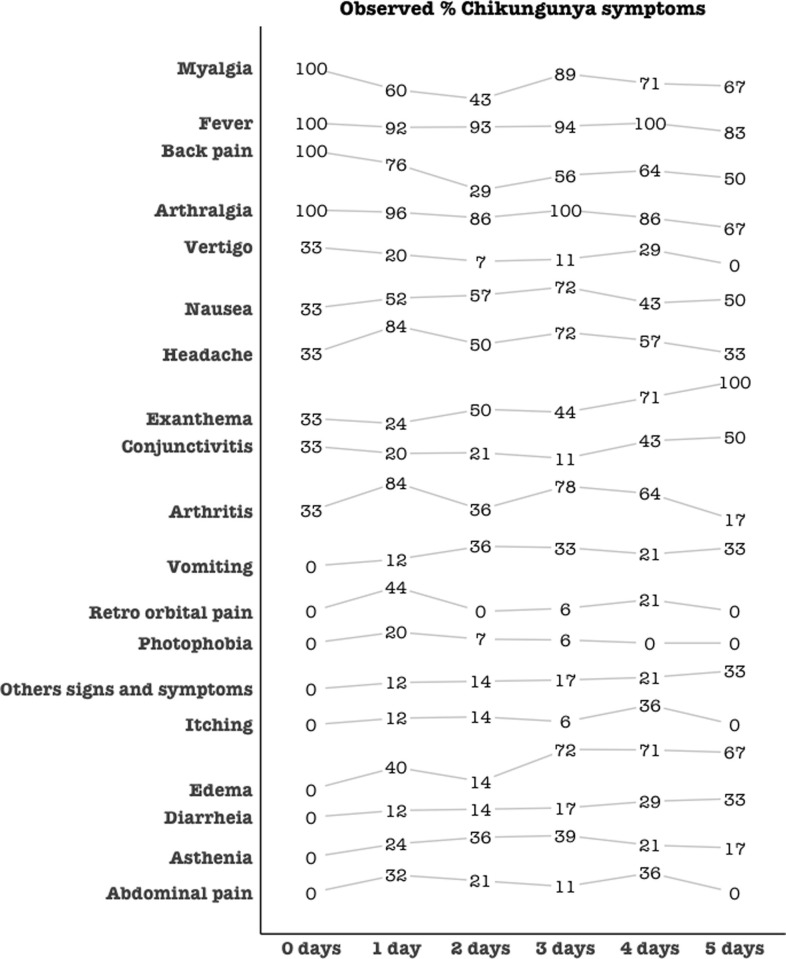
Chikungunya signs and symptoms. Temporal sequence of clinical signs and symptoms in acute chikungunya infection cases.

Myalgia, fever, back pain, and arthralgia were among all signs and symptoms described on the first day of symptom onset as the most prevalent (100%), but only fever and arthralgia remained fairly constant and high, even for more than 5 days of symptoms (71% and 86%, respectively) (Fig 5). The complaint of exanthema, diarrhea and edema gradually increased after 2 or 3 days after the onset of symptoms (Fig 5).

## Discussion

CHIKV is an emerging arbovirus which causes severe public health problems [21, 22] and can lead to important loss of disability-adjusted life-years (DALY) [23, 24]. The introduction of the virus in Brazil is of great concern since the tropical climate is favorable for the two main mosquito vectors: *Aedes aegypti* and *Aedes albopictus*. Surveillance and control are difficult because these mosquitoes are widespread throughout the country [25, 26]. In addition, the co-circulation of other arboviruses such as dengue and zika viruses also make the clinical diagnosis and the therapeutic approach difficult [21, 27, 28].

In agreement with preview arbovirosis work [29, 30], we found the highest number of positive cases of CHIKV infection in the first 3 months of 2016 (January to March) and the peak was in March. The pluviometric index is an important factor for the increase in vector population as the female *A*. *aegypti* needs water to lay eggs [31], and therefore the effect of the rainfall index and the incidence of CHIKV cases during the 2016 outbreak were evaluated. Differently from the Rio de Janeiro outbreak [30], the Natal precipitation increase does not precede the CHIKV transmission peak, and therefore it does not seem to be an important factor in the local transmission dynamics.

The qRT-PCR data show a higher number of infected women, compared to men. In another hand, men presented higher chance to be infected. These gender differences are very dependent to community-specific habits, customs or behaviors. It is important to remember the zika virus epidemics occurred in a previous year in the studied area, so we should keep in mind that the health services are very careful with women, especially those in a fertile age. In addition, women are more likely to seek medical care than males. This could explain the greater amount of women studied.

The present work found that the age group of older than 61 years was one of the most affected by the chikungunya virus. This age group may also be considered more susceptible to disease. In addition, we also found that adults aged between 41 to 50 years were highly affected by chikungunya infection during the studied period. The incidence was similarly higher in persons older than 40 years old in the Rio de Janeiro chikungunya outbreak [30].

Chikungunya viremia is influenced by the symptom onset time [32, 33]. Our data also showed a gradual decrease of viremia over the days of symptom onset. Although 5 days after symptom onset is characterized by the very low or absence of viremia, we were also able to detect CHIKV in 10 volunteers, even 23 days after symptom onset. CHIKV RNA was detected in many types of samples; however, serum and WB presented the highest viral load, probably because the samples were collected exactly in the critical period of viremia. However, we should keep in mind that this comparison is difficult due to the lack of different types of samples from the same patient collected on the same day. The present study could not find a correlation between age and CHIKV viremia, as previously observed [33].

The incubation period of CF is followed by fever and an intense diffuse myalgia and joint pain [24, 34]. More than 50% of positive CHIKV volunteers presented the following clinical manifestations: fever, arthralgia, myalgia, arthritis, headache and back pain. Although the most prevalent complaint of patients was joint pain, a significant number of the female subjects presented abdominal pain compared to men. The mechanism of non-articular pain associated with CHIKV infection is still poorly understood, however females seems to be more affected by pain [34].

Acute CF is mainly characterized by fever and arthralgia, followed by a decrease in its symptom complaint (>5days) [35, 36]. On the other hand, the percentage of both symptoms remained elevated even after 5 days of symptom onset. The positive CHIKV volunteers complained about myalgia and exanthema even in the very early days of symptom onset, which is different from an Indian study [35, 36]. Therefore, different CHIKV outbreak could exhibit a distinct pattern of clinical manifestations.

Skin blistering could be observed in our data as previously described for outbreaks in different areas [37, 38]. This dermatologic manifestation was described for 10 subjects, and 8 of them presented positive PCR. Among these subjects, 4 were < 1 year old and 4 were adult (including 2 pregnant women). Although skin blistering is considered a differential diagnosis for CHIKV [39], 2 subjects presented negative qRT-PCR using serum as sample. We found 100% of the analyzed blister fluid by CHIKV-PCR positive, and therefore we encourage the use of this sample in qRT-PCR diagnosis. This sign was grouped as ¨other signs and symptoms” based on the low frequency of blistering. All symptoms described herein are in agreement with Brazil Health Ministry description, as well as studies developed in other countries [12, 40, 41].

Among all the clinical signs and symptoms, fever, arthralgia and conjunctivitis were more common among the positive CHIKV group. This information can be important to physicians during a clinical evaluation of suspected CHIKV cases.

In order to investigate the possibility of a Flavivirus infection in a patient with a chikungunya clinical diagnosis, we also evaluated the presence of anti-CHIKV and anti-FV IgM in 120 CHIKV suspected and negative qPCR cases. As part of the flavivirus family, ZIKV and DENV1-4 share similar genetic and structural characteristics, and show high homology with at least 51 to 53% of amino acid identity [42, 43]. NS1 is the main antigen used to identify anti-ZIKV and anti-DENV1-4 antibodies in serological protocols, but this high similarity between antigens can lead to cross-reaction and low specificity [44]. Therefore, we opted to represent ZIKV and DENV1-4 as a unique FV group. CHIKV and ZIKV/DENV1-4 belong to different families and do not share similar structures or antigens. Thus, we considered the double positive samples as co-exposition probable cases.

Due to the entrance and spread of ZIKV in the Brazilian territory, the co-circulation of CHIKV, ZIKV and DENV1-4 was established in Brazil after 2015. Co-circulation of the three arboviruses was identified in northeastern Brazil [45, 46]. Confirming the difficulty of an accurate viral clinical diagnosis, we found 21.7% positive for IgM flavivirus in Chikungunya suspected cases.

Serological and molecular evidence of FV infection after previous CHIKV exposition was also found in patients with suspected acute arbovirus infection in Campo Grande city, in the Mid-west region of Brazil [45]. Similarly, in the present study we found immunological evidence of CHIKV/FV co-circulation in Natal, Rio Grande do Norte state, which indicates the occurrence of concomitant exposition to these viruses during the 2016 CHIKV outbreak.

## Conclusion

All the findings discussed above can contribute to increase knowledge about the clinical-epidemiological characteristic of chikungunya virus transmission in Brazil, and could be a useful tool in preventing a future outbreak, as well as helping in the clinical diagnosis of chikungunya virus infection.

## Supporting information

S1 Fig Validation of anti-CHIKV and anti-FV IgM ELISA protocols. Receiver Operating Characteristic (ROC) curves to anti-CHIKV, anti-ZIKV and anti-DENV1-4 IgM ELISA protocols. Data in A-F are presented as sensitivity and specificity percentages, 95% CI. Dot lines represent identity lines of curves.
(TIF)

S2 Fig Chikungunya virus detection in different age groups. Absolute number and Positivity of chikungunya virus between age groups, during the outbreak in the Rio Grande do Norte, Brazil, 2016.
(TIF)

S3 Fig Skin blistering in chikungunya case. Skin blisters in neonates with chikungunya fever during the outbreak in the State of Rio Grande do Norte, Brazil, 2016.
(TIF)

S1 Table Receiver Operating Characteristic (ROC) analysis to anti-CHIKV and anti-FV IgM ELISA protocols. (DOCX)
