# Supplementary figures and images for: Epidemiologic and clinical investigations during a chikungunya outbreak in Rio Grande do Norte State, Brazil
Source: PLoS One. 2020 Nov 20;15(11):e0241799. doi: 10.1371/journal.pone.0241799 (PMC7678967; doi:10.1371/journal.pone.0241799)

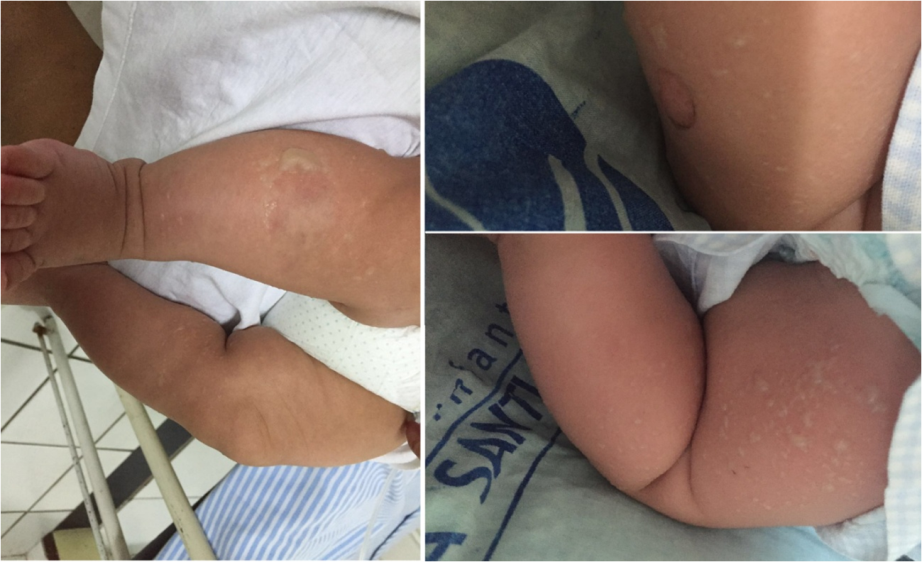

Supplement: S3 Fig — Skin blisters in neonates with chikungunya fever during the outbreak in the State of Rio Grande do Norte, Brazil, 2016. (TIF) [file pone.0241799.s003.tif]
