# Supplementary material for: Epidemiologic and clinical investigations during a chikungunya outbreak in Rio Grande do Norte State, Brazil
Source: PLoS One. 2020 Nov 20;15(11):e0241799. doi: 10.1371/journal.pone.0241799 (PMC7678967; doi:10.1371/journal.pone.0241799)
Supplement: S1 Table — (DOCX) [file pone.0241799.s004.docx]

**S1 Table. Receiver Operating Characteristic (ROC) analysis to anti-CHIKV and anti-FV IgM ELISA protocols.**

| **ELISA protocol** | **AUC value** | **AUC range (95% CI)** | **p-value** | **rOD threshold** | **Sensitivity (%)** | **Specificity (%)** |
| --- | --- | --- | --- | --- | --- | --- |
| Anti-CHIKV IgM | 0.9705 | 0.9368 to 1.0004 | <0.0001 | 1.126 | 100.00 | 49.23 |
| Anti-ZIKV IgM | 0.7342 | 0.6148 to 0.8537 | 0.0003 | 1.112 | 89.66 | 15.38 |
| Anti-DENV1 IgM | 0.5224 | 0.3562 to 0.6886 | 0.7914 | 1.219 | 85.00 | 10.34 |
| Anti-DENV2 IgM | 0.5310 | 0.3657 to 0.6964 | 0.7143 | 1.100 | 90.00 | 13.79 |
| Anti-DENV3 IgM | 0.5276 | 0.3629 to 0.6923 | 0.7448 | 1.195 | 90.00 | 13.79 |
| Anti-DENV4 IgM | 0.5293 | 0.3647 to 0.6939 | 0.7295 | 1.140 | 95.00 | 10.34 |

AUC: area under the curve; rOD: relative optical density.
